# Use of Perfluorohexyloctane for Preservation of Rat Liver After Circulatory Death and a Prolonged Cold Preservation Model for Hepatocyte Transplantation

**DOI:** 10.3389/ti.2022.10659

**Published:** 2022-07-05

**Authors:** Muneyuki Matsumura, Takehiro Imura, Akiko Inagaki, Hiroyuki Ogasawara, Shigehito Miyagi, Kazuo Ohashi, Michiaki Unno, Takashi Kamei, Masafumi Goto

**Affiliations:** ^1^ Department of Surgery, Tohoku University Graduate School of Medicine, Sendai, Japan; ^2^ Division of Transplantation and Regenerative Medicine, Tohoku University School of Medicine, Sendai, Japan; ^3^ Graduate School and School of Pharmaceutical Sciences, Osaka University, Osaka, Japan

**Keywords:** donation after circulatory death, preservation solution, perfluorohexyloctane, hepatocyte transplantation, prolonged cold preservation model

Dear Editors,

Liver transplantation has been established as the standard therapy for end-stage liver disease, and hepatocyte transplantation (HTx) has been gaining acceptance as an alternative for the treatment of patients with inherited metabolic liver diseases and acute hepatic failure. However, due to donor shortage, the main tissue sources for HTx are marginal-quality livers (i.e., livers donated after circulatory death, those exposed to prolonged cold storage, etc.). In the field of pancreatic islet transplantation, which is a cell therapy similar to HTx, pancreas oxygenation using perfluorohexyloctane (F6H8) has been shown to effectively prevent ischemically induced damage incurred during cold preservation [1–3]. F6H8 has a high lipophilic character compared to conventionally used perfluorodecaline, subsequently resulting in a high oxidizing capacity for the graft. Therefore, the present study assessed whether or not hepatocyte isolation using livers from donation after circulatory death and prolonged cold ischemic time could be improved using F6H8.

Male F344/NSLc rats were anesthetized and systemically heparinized, and then warm ischemia was induced by incising the diaphragm. The heart stopped beating within approximately 6 min of starting the procedure. The period of warm ischemic time was 15 min, and then the portal vein was cannulated. University of Wisconsin (UW) solution was flushed *via* the portal vein. Grafts were assigned to 2 groups: those preserved in UW solution (UW group) or in the oxygenated F6H8 (F6H8 group) at 4°C for 72 h. Therefore, cold ischemic time in this experiment was 72 h. F6H8 was oxygenated with gaseous oxygen for 10 min before use. After preservation, in both groups, the hepatocytes were isolated and purified using a modified two-step collagenase perfusion technique as previously described [4]. Hepatocyte viability was evaluated by trypan blue exclusion (TBE) and the ADP/ATP ratio [5, 6]. Ten million hepatocytes were then directly injected into the portal vein of analbuminemic rats [7], and the serum albumin levels were quantified on days 0, 14, and 28. All animals were handled according to the Guide for the Care and Use of Laboratory Animals, and the guidelines for animal experiments at Tohoku University (protocol ID: 2014 NICHe-Animal-001).

The TBE viability of the UW group was significantly higher than that of the F6H8 group (76.50 ± 3.77% and 70.53 ± 5.59%, *n* = 8, *p* = 0.025). In terms of the ADP/ATP ratio, no significant difference was observed between groups (UW: 0.175 ± 0.057, F6H8: 0.149 ± 0.046, *n* = 8, *p* = 0.329). In terms of the serum albumin levels, no significant difference was observed between the UW (day 0: 6.13 ± 1.24 μg/ml, day 14: 6.33 ± 2.90 μg/ml, day 28: 6.16 ± 1.31 μg/ml, *n* = 10) and F6H8 (day 0: 5.56 ± 1.33 μg/ml, day 14: 10.34 ± 10.06 μg/ml, day 28: 5.64 ± 0.69 μg/ml, *n* = 10) (*p* = 0.19) groups (Table 1). The TBE and ADP/ATP ratio were analyzed by Student’s t-test, and the serum albumin levels were analyzed by a two-way analysis of variance.

**TABLE 1:** The results of Cell yield, TBE viability, ADP/ATP ratio, serum albumin levels on days 0, on days 14 and on days 28 in UW group and F6H8 group.

	Cell Yield (10^8^ cells)	TBE viability (%)	ADP/ATP Ratio	Serum albumin levels on days 0 (µg/ml)	Serum albumin levels on days 14 (µg/ml)	Serum albumin levels on days 28 (µg/ml)
UW group	4.90 ± 1.10	76.50 ± 3.77	0.175 ± 0.057	6.13 ± 1.24	6.33 ± 2.90	6.16 ± 1.31
F6H8 group	4.49 ± 0.77	70.53 ± 5.59	0.149 ± 0.046	5.56 ± 1.33	10.34 ± 10.06	5.64 ± 0.69

TBE, trypan blue exclusion; UW, University of Wisconsin, F6H8 = perfluorohexyloctane.

Unexpectedly, the strategy for preventing ischemically induced damage in marginal graft model using oxygenated F6H8 compared to conventional storage of UW does not seem to improve the outcomes of HTx. We found TBE viability of the UW group was significantly higher than that of the F6H8 group. But in terms of the ADP/ATP ratio and serum albumin levels, we could not find any statistically significant differences between both groups, suggesting that only a limited increase of necrotic (but not apoptotic) hepatocytes might be observed in the F6H8 group for unknown reasons, but this difference was too small to be reflected in the transplant outcomes. In this experiment, the warm ischemic time and cold ischemic time were longer than those in real clinical settings. We performed a preliminary experiment with a shorter cold ischemic time, but no significant differences were observed.

In conclusion, UW solution, in comparison to the F6H8, may have better cytoprotective effect on preserving liver tissues from marginal-quality donors. However, this effect was not enough to facilitate the engraftment of hepatocytes. More robust approaches to improve the quality of liver grafts are needed for HTx using marginal-quality donors.

## Data Availability

The original contributions presented in the study are included in the article/supplementary material, further inquiries can be directed to the corresponding author.
